# Association between overall diet quality and postmenopausal breast cancer risk in five Finnish cohort studies

**DOI:** 10.1038/s41598-021-95773-2

**Published:** 2021-08-18

**Authors:** Satu Männistö, Kennet Harald, Tommi Härkänen, Mirkka Maukonen, Johan G. Eriksson, Sanna Heikkinen, Pekka Jousilahti, Niina E. Kaartinen, Noora Kanerva, Paul Knekt, Seppo Koskinen, Maarit A. Laaksonen, Nea Malila, Harri Rissanen, Janne Pitkäniemi

**Affiliations:** 1 Finnish Institute for Health and Welfare, PO Box 30, 00271 Helsinki, Finland; 2 Department of General Practice and Primary Health Care, University of Helsinki and Helsinki University Hospital, Helsinki, Finland; 3 Folkhälsan Research Center, Helsinki, Finland; 4 Singapore Institute for Clinical Science, Agency for Science, Technology, and Research, Singapore, Singapore; 5 Department of Obstetrics and Gynaecology, Yong Loo Lin School of Medicine, Human Potential Translational Research Programme, National University of Singapore, Singapore, Singapore; 6 Finnish Cancer Registry, Institute for Statistical and Epidemiological Cancer Research, Helsinki, Finland; 7 School of Mathematics and Statistics, University of New South Wales, Sydney, Australia; 8 Department of Public Health, University of Helsinki, Helsinki, Finland; 9 School of Health Sciences, University of Tampere, Tampere, Finland

**Keywords:** Diseases, Risk factors

## Abstract

There is limited evidence for any dietary factor, except alcohol, in breast cancer (BC) risk. Therefore, studies on a whole diet, using diet quality indices, can broaden our insight. We examined associations of the Nordic Diet (mNDI), Mediterranean diet (mMEDI) and Alternative Healthy Eating Index (mAHEI) with postmenopausal BC risk. Five Finnish cohorts were combined including 6374 postmenopausal women with dietary information. In all, 8–9 dietary components were aggregated in each index, higher total score indicating higher adherence to a healthy diet. Cox proportional hazards regression was used to estimate the combined hazard ratio (HR) and 95% confidence interval (CI) for BC risk. During an average 10-year follow-up period, 274 incident postmenopausal BC cases were diagnosed. In multivariable models, the HR for highest vs. lowest quintile of index was 0.67 (95 %CI 0.48–1.01) for mNDI, 0.88 (0.59–1.30) for mMEDI and 0.89 (0.60–1.32) for mAHEI. In this combined dataset, a borderline preventive finding of high adherence to mNDI on postmenopausal BC risk was found. Of the indices, mNDI was more based on the local food culture than the others. Although a healthy diet has beneficially been related to several chronic diseases, the link with the etiology of postmenopausal BC does not seem to be that obvious.

## Introduction

Breast cancer (BC) is the most common female malignancy accounting for 1 in 4 cancer cases worldwide^1^. Furthermore, it is the leading cause of cancer death in women. In all, about 70% of BC cases are diagnosed in women aged 50 years or over^2^. Genetic, hormonal and reproductive factors as well as lifestyle-related factors, such as obesity and exercise, are associated with BC risk^3^.

The etiology of BC may differ by menopausal status as a consequence of varying effects of diet on hormones in different stages of life^3^. The systematic review by World Cancer Research Fund^3^, however, summarized that there is only limited evidence between any foods or nutrients, except alcohol, and premenopausal or postmenopausal BC risk. Evaluating one dietary factor at a time does not take into account the complexity of human diet, which includes a large number of candidate factors and their interactions. In all, diet as a whole may have a greater effect on health than any specific foods or nutrients, and thus, considering the whole diet gives a broader perspective for developing public health recommendations.

A systematic review including seven cohort studies concluded that the adherence to the Mediterranean diet decreased BC risk by 6%^4^. Findings for less examined diet quality indices, such as the Healthy Eating Index (HEI), Alternative Healthy Eating Index (AHEI) and Dietary Approaches to Stop Hypertension (DASH), have been inconclusive^5–9^. In a Swedish cohort study (n = 44,296 women aged 29–49 years at baseline), adherence to the Healthy Nordic Food Index was not associated with premenopausal or postmenopausal BC risk in the Uppsala Health Care Region^10^.

The aim of this study was to examine associations between adherence to three diet quality indices, based on Nordic, Mediterranean and American dietary recommendations, and postmenopausal BC risk in a combined dataset of five Finnish cohort studies.

## Subjects and methods

### Subjects

The Prospective Meta-Cohort Study of Cancer Burden in Finland (METCA project) aims to examine the determinants of cancer burden involving multiple population-based cohort studies and utilizing national health registries^11^. Of those, five cohorts were included in the present BC study (Table 1): Finnish Mobile Clinic Follow-up Survey (FMCF)^12^, Health 2000 Survey (Health 2000)^13^, Helsinki Birth Cohort Study (HBCS)^14^, National FINRISK Study 2007 (FINRISK 2007)^15^ and National FINRISK 2012 Study (FINRISK 2012)^16^. In this present study, we included women at least aged 50 years (n = 7720) and who filled in acceptable long-term dietary information (83%). Thus, the final data consisted of 6374 postmenopausal women.

**Table 1:** Cohort studies included in the combined analyses of the association between diet quality indices and postmenopausal breast cancer risk.

Cohort studies	Baseline and the end year of follow-up	Women aged 50 + years	Women aged 50 + years with FFQ^a^ (% 50 + years women)	Follow-up time (median, years)	Number of breast cancer cases
Finnish Mobile Clinic Follow-up Survey (FMCF)^12^	1973–1976 to 2013	1126^b^	1115 (99%)	24.9	46
Health 2000 Survey (Health 2000)^13^	2000–2001 to 2015	2148^c^	1639 (76%)	15.1	86
Helsinki Birth Cohort Study (HBCS)^14^	2001–2004 to 2014	1075^d^	981 (91%)	12.0	65
National FINRISK 2007 Study (FINRISK 2007)^15^	2007 to 2016	1555^e^	1327 (85%)	9.6	45
National FINRISK 2012 Study (FINRISK 2012)^18^	2012 to 2016	1816^c^	1312 (72%)	4.8	32
Total	–	7720	6374^f^ (83%)	9.7	274

^a^Food frequency questionnaire.

^b^Dietary subgroup with a dietary history interview.

^c^Food frequency questionnaire was given for those who participated in health examination at study site.

^d^Clinical subgroup.

^e^Dietary subgroup with a food frequency questionnaire.

^f^77% of women aged 50 + years had accepted FFQ.

Each cohort study included a health examination with measures and blood samples and a health questionnaire with questions, for example, on socioeconomic status, lifestyle, reproductive factors and medical history. These variables were harmonised across the cohort studies as shown in the tables. Weight and height were measured with light clothing and without shoes by trained nurses at study sites. Body mass index (BMI) was calculated as a participant's weight in kilograms divided by the square of height in meters (kg/m^2^).

This study was performed in line with the principles of the Declaration of Helsinki. All cohort studies followed the code of ethics in effect at the time of the study. In the more recent cohort studies, for example, all procedures involving participants were approved by the Ethics Committee of Helsinki and Uusimaa Hospital District. Written informed consent was obtained from the participants.

### Dietary assessment

The habitual diet was assessed by a dietary history interview method (FMCF)^17^ or a food frequency questionnaire (FFQ, other cohort studies) developed and updated at the Finnish Institute for Health and Welfare (THL)^18–20^.

In FMCF, the dietary history interview method covered the habitual diet over the last 12 months^17^. The structured interview was carried out at the study site with a questionnaire including more than 100 foods. The food models were used to assess an average portion size a day, week, month or year. The daily food consumption and nutrient intakes were calculated using the food composition database of the Social Insurance Institution^21^. The short-term and long-term reproducibility of the dietary method was found to be acceptable and sufficiently stable for the needs of epidemiological studies^17^.

In the other cohort studies, a semi-quantitative FFQ was used to assess the habitual diet over the last 12 months^22^. The FFQ has been updated every five years since 2000 based on the National Findiet Survey^23^ carried out by THL. The food rows of FFQ (128–131 rows) have remained largely unchanged and the updates have concerned the sex-specific portion sizes and the food composition database codes composing of the food rows^22^. The average consumption of each food was recorded by nine frequency categories ranging from ‘never or seldom’ to ‘at least six times a day’. The FFQ was given to all participants in the health examination and they were asked to complete it at the study site or at home. The FFQ data were converted into average daily food consumption and nutrient intakes using the National Food Composition Database, Fineli and the Finessi software of THL^24^. The previous reproducibility^18,19^ and validation studies^18–20^ have shown the FFQ to be acceptable for the purpose of epidemiological studies.

### Diet quality indices

Three diet quality indices, the modified Nordic Dietary Index (mNDI), modified Mediterranean Diet index (mMEDI) and modified Alternate Healthy Eating Index (mAHEI), were utilized to describe the study-specific adherence to a healthy diet (Supplementary Table S1). In all, 8–9 food or nutrient components were aggregated in each index, higher score indicating higher adherence to a healthy diet. All the indices were slightly modified from the original ones to be more in line with the current Nordic dietary recommendations^25^.

The NDI, consisting of nine dietary components, was originally developed and validated by Kanerva et al.^26^. These components included fruits; vegetables; whole-grain products; low-fat milk; fish; red and processed meat; total fat intake (E%); a ratio of polyunsaturated fatty acids to saturated fatty acids and trans-fatty acids; and alcohol. In food components, the emphasis was on fruits, vegetables and grains that can be cultivated in the Nordic countries as well as domestic fish. Total fat intake, however, was excluded from the mNDI (Supplementary Table S1). All components, except alcohol, were scored from 0 to 3 points based on the quartiles of sex-specific intakes. The points were related to predictable health impact of the component. Fox example, for fruits the lowest intake quartile was coded as 0 and the highest as 3 while the coding was the reverse for red and processed meat. Alcohol consumption of no more than 20 g/day in men and 10 g/day in women (calculated as ethanol) was scored as 1 point and otherwise 0 points. The score range for the mNDI was 0–22 points.

The mMEDI was based on the Mediterranean Diet Index by Trichopoulou et al.^27^ and its modification by Fung et al.^5^. The original index included ten components: Fruits and nuts; vegetables; legumes; cereals; dairy; fish; meat and meat products; poultry; a ratio of monounsaturated fatty acids to saturated fatty acids; and alcohol^27^. We used the index modified by Fung et al.^5^ as follows: separating fruits and nuts into two groups, excluding potato from the vegetable group, including only whole-grain products, excluding the dairy group and including only red and processed meat (Supplementary Table S1). Those participants whose intake was above the median intake received 1 point while others received 0 points with two reverse exceptions (meat and alcohol). The score range for the mMEDI was 0–9 points.

The mAHEI was adapted from the method by McCullough et al.^28^ including nine components: fruits; vegetables; nuts and soy; cereal fibre; a ratio of white meat to red meat; a ratio of polyunsaturated fatty acids to saturated fatty acids; trans-fatty acids; alcohol; and multivitamin use. We modified the original index by replacing fibre from grain sources by whole-grain products, excluding multivitamin use (not recommended in the Nordic dietary recommendation), and in general, using grams per day instead of servings for food and alcohol components (Supplementary Table S1). We also modified the original score calculation (intermediate intakes were scored proportionately between 0 and 10) so that all components, except alcohol, were scored from 1 to 5 points based on the quintiles of sex-specific intakes^29^. For example, for fruits the lowest intake quintile was coded as 1 and the highest as 5 while the coding was the reverse for trans-fatty acids. Furthermore, men consuming no more than 20 g and women 10 g alcohol (calculated as ethanol) per day received 1 point while 0 points was given to the others. The score range for the mAHEI was 7–36 points.

### Breast cancer cases

Incident BC cases (ICD-10 code C50) were obtained from the Finnish Cancer Registry, which includes national cancer data with high reliability and comprehensiveness since 1953^30^. The participants of each cohort study were linked to the register through a unique personal identity code issued to each Finnish citizen or permanent resident. The follow-up periods of cohort studies are presented in Table 1. All women who had diagnosed BC or any other cancer before baseline were excluded. All incident BC cases were assumed to have postmenopausal BC.

### Statistical methods

The combined data set of five cohort studies with harmonized dietary factors and covariates were used in the statistical analyses. Hazard ratios (HR) and 95% confidence intervals (CI) for BC according to the quintiles of diet quality indices (scores) or their dietary components (intakes) were calculated using one-stage mixed effect Cox proportional models, as the number of BC cases was small in the individual studies^31^. In each study, person-years of follow-up were calculated from the date of the health examination visit at baseline to the date of BC diagnosis, the date of death, or the end of follow-up, whichever came first. The simple regression model was adjusted for age. The multivariable regression model was further adjusted for education, smoking, height, BMI, leisure time exercise, parity, hormone replacement therapy, energy intake and cohort. We carried out sensitivity analyses in which we (i) additionally included type 2 diabetes (T2D) as a potential confounding factor (ii) used the attained age as the time scale instead of follow-up years (iii) excluded the incident BC cases during the first two years of follow-up while undiagnosed cancer might affect eating habits and iv) excluded the alcohol component (obvious risk factor of BC in the previous literature) from each index and included it in the multivariable models as a covariate. The proportional hazard assumption was tested using the Schoenfeld residuals. Tests for linear trends across the quintiles of diet quality indices were performed using the Wald test by modelling the median value of each quintile as a continuous variable. The heterogeneity between studies as well as categories of BMI, leisure time exercise and follow-up period were tested using the type 3 test of the interaction coefficients of cohort/category and diet quality index implemented in the R package car^32^. All p values were two sided, and p values less than 0.05 were considered statistically significant. The analyses were performed using R version 3.6.0^33^ statistical programs.

### Ethics declarations

This study was performed in line with the principles of the Declaration of Helsinki. All cohort studies followed the code of ethics in effect at the time of the study. In the more recent cohort studies, for example, all procedures involving participants were approved by the Ethics Committee of Helsinki and Uusimaa Hospital District.

### Consent to participate

Informed signed consent was obtained from all individual participants included in the study.

## Results

The combined data included 274 incident postmenopausal BC cases from 6374 Finnish women aged 50 years and over who were followed for median 5–25 years across the cohort studies (Table 1). At baseline, the median age of the participants ranged from 57 (FMCF) to 63 years (Health 2000; Table 2). The women from HBCS were more likely to be current smokers, more exercise at leisure time and more likely to use hormone replacement therapy compared to women from the other cohorts, except for FINRISK 2012 in which the leisure time exercise was also high. The median BMI was 27.0 kg/m^2^ across the cohorts and 85% of the women had given birth.

**Table 2:** Characteristics (median/%) of women included in the combined analysis of the association between dietary indices and postmenopausal breast cancer risk.

	Cohort studies				
	FMCF	Health 2000	HBCS	FINRISK 2007	FINRISK 2012
Age, years (SD)	57 (5.9)	63 (10.9)	60 (3.1)	61 (6.9)	62 (6.8)
High education^a^, %	3	35	32	36	34
Current smoker, %	8	13	21	12	13
Height, cm (SD)	158 (5.7)	160 (6.4)	163 (5.8)	161 (5.9)	162 (5.9)
Body mass index, kg/m^2^ (SD)	26.7 (4.4)	27.4 (4.9)	26.9 (5.0)	26.6 (5.2)	26.9 (5.3)
Leisure time exercise^b^, %	5	11	24	19	25
Parity, %	84	88	87	87	86
Hormone replacement therapy, ever%	0^c^	47	69	27	26
mNordic Dietary Index, points (SD)	11 (3.1)	12 (3.6)	11 (3.7)	12 (3.7)	13 (3.5)
mMediterranean Dietary Index, points (SD)	4 (1.5)	4 (1.8)	4 (1.9)	5 (1.8)	5 (1.9)
mAlternative Healthy Eating Index, points (SD)	21 (4.1)	22 (4.8)	21 (4.9)	22 (4.7)	23 (4.9)

^a^The total number of school years was divided into birth cohort specific tertiles with an exception of the Finnish Mobile Clinic Follow-up Survey where ‘highest education’ included those with more than upper secondary education.

^b^Active, ≥ 3 times per week.

^c^Hormone replacement therapy was not asked in Finnish Mobile Clinic Follow-up Survey. It was assessed to be ‘not used’ for all women as the treatment was rare in Finland in the 1970s^45^.

The median scores of diet quality indices at baseline were slightly higher in the more recent cohorts than the oldest ones: from 11 to 13 points for mNDI, from 4 to 5 points for mMEDI and from 21 to 23 points for mAHEI (Table 2). Furthermore, the average consumption of whole-grain products and red and processed meat were lower whereas the consumption of the other components (e.g., fruits, vegetables, and fish) was higher in the most recent cohorts compared to the others (Supplementary Table S2). In FINRISK 2012, the intake of saturated fatty acids was one-third lower, and intake of polyunsaturated fatty acids 2 times higher than in FMCF.

**Figure 1:**
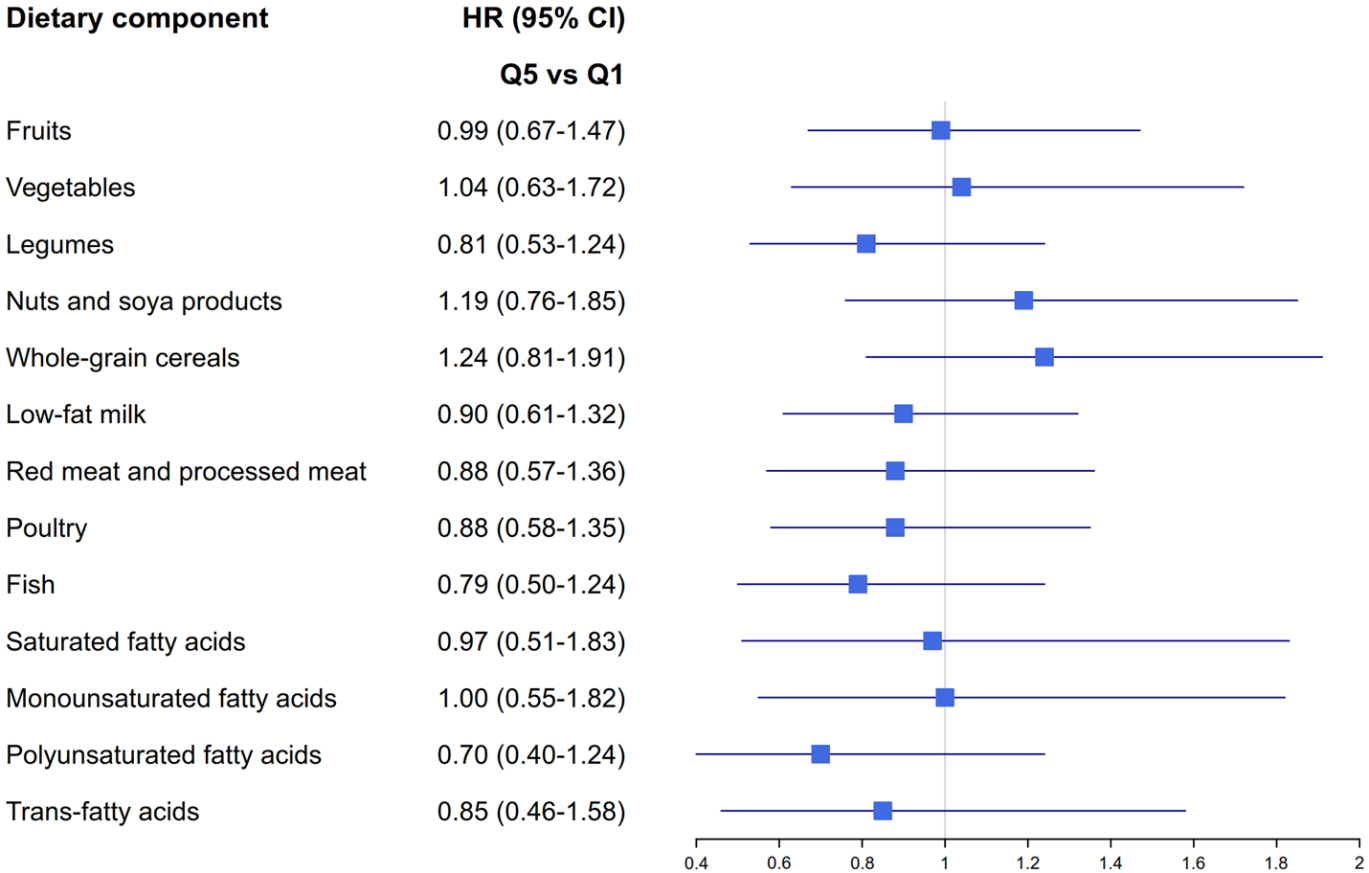
Multivariable hazard ratios (HR) and confidence intervals (95% CI) of postmenopausal breast cancer by highest (Q5) and lowest (Q1) of specific dietary index components.

In the multivariable model, the HR of BC for the highest compared to the lowest quintile of diet quality index was 0.67 (95% CI 0.48–1.01, test for trend: p = 0.18) for mNDI, 0.88 (95% CI 0.59–1.30, test for trend: p = 0.27) for mMEDI and 0.89 (95% CI 0.60–1.32, test for trend: p = 0.47) for mAHEI (Table 3). When the proportional hazard assumption was tested using the Schoenfeld residuals, the global p-values were not statistically significant (p = 0.76 for mNDI, p = 0.73 for mMEDI and p = 0.77 for mAHEI), and thus, the assumption was not violated. The results were similar when using the attained age instead of follow-up years as the underlying time scale in sensitivity analyses, the hazard ratio between the highest compared to the lowest quintile of diet quality index was 0.69 (95% CI 0.46–1.05, test for trend: p = 0.21) for mNDI, 0.98 (95% CI 0.67–1.45, test for trend: p = 0.65) for mMEDI and 0.95 (95% CI 0.64–1.41, test for trend: p = 0.70) for mAHEI. The results were also similar when additionally adjusted for T2D. The results attenuated slightly when incident BC cases during the first two follow-up years (n = 53) were excluded, except for mMEDI. When the alcohol component was excluded from each index and included in the multivariable models as a covariate, the HR of BC for the highest compared to the lowest quintile of diet quality index was 0.70 (95% CI 0.45–1.02, test for trend: p = 0.21) for mNDI, 0.85 (95% CI 0.57–1.26, test for trend: p = 0.25) for mMEDI and 0.82 (95% CI 0.56–1.21, test for trend: p = 0.29) for mAHEI. There was no evidence of heterogeneity of the association of diet quality indices with BC risk between study cohorts. Furthermore, the associations between the diet quality indices and BC risk were not modified by the categories of BMI, leisure time exercise or follow-up period (Table 4). No associations were found between the dietary components of indices and BC risk (Fig. 1), although the intake ranges were wide (Supplementary Table S2).

**Table 3:** Hazard ratios (HR) and confidence intervals (95% CI) of postmenopausal breast cancer by quintiles of dietary indices.

	Quintile 1	Quintile 2	Quintile 3	Quintile 4	Quintile 5	P_trend_^c^	P_between-studies heterogeneity_^d^
**mNordic Dietary Index**							
Index points, median (SD)	7 (1.5)	10 (0.7)	12 (0.7)	14 (0.7)	17 (1.4)		
Breast cancer cases, n	56	54	57	62	45		
Age-adjusted	1.00	0.97 (0.67–1.41)	1.03 (0.71–1.49)	1.08 (0.75–1.55)	0.76 (0.51–1.13)	0.35	0.15
Multivariable^a^	1.00	0.95 (0.65–1.38)	1.03 (0.71–1.49)	1.05 (0.72–1.51)	0.67 (0.48–1.01)	0.18	0.14
Multivariable^b^	1.00	1.19 (0.78–1.82)	1.17 (0.76–1.79)	1.18 (0.77–1.80)	0.83 (0.52–1.33)	0.50	0.31
**mMediterranean Dietary Index**							
Index points, median (SD)	2 (0.7)	3 (0.5)	4 (0.5)	5 (0.5)	7 (0.8)		
Breast cancer cases, n	59	56	47	50	62		
Age-adjusted	1.00	0.94 (0.65–1.35)	0.77 (0.52–1.12)	0.82 (0.56–1.20)	1.03 (0.72–1.47)	0.88	0.29
Multivariable^a^	1.00	0.89 (0.61–1.28)	0.72 (0.49–1.07)	0.69 (0.46–1.02)	0.88 (0.59–1.30)	0.27	0.24
Multivariable^b^	1.00	0.91 (0.61–1.36)	0.63 (0.40–0.99)	0.69 (0.45–1.06)	0.87 (0.56–1.34)	0.27	0.23
**mAlternative Healthy Eating Index**							
Index points, median (SD)	16 (1.9)	19 (0.9)	22 (0.8)	25 (0.9)	28 (2.0)		
Breast cancer cases, n	54	58	57	52	53		
Age-adjusted	1.00	1.01 (0.70–1.47)	1.05 (0.72–1.52)	0.95 (0.65–1.39)	0.96 (0.65–1.40)	0.73	0.31
Multivariable^a^	1.00	1.02 (0.71–1.49)	1.00 (0.69–1.46)	0.93 (0.63–1.38)	0.89 (0.60–1.32)	0.47	0.29
Multivariable^b^	1.00	1.17 (0.76–1.78)	1.21 (0.79–1.85)	0.99 (0.63–1.55)	1.03 (0.66–1.61)	0.80	0.21

^a^Multivariable model was adjusted for age (years), education (tertiles by birth year), smoking (never, former, current smokers), height (cm, continuous), body mass index (kg/m^2^, continuous), leisure time exercise (passive, somewhat active, active), parity (never, ever), hormone replacement therapy (never, ever), energy intake (kJ/day, continuous) and cohort.

^b^Excluding cases diagnosed during the first two years of follow-up: Finnish Mobile Clinic Follow-up Survey (n = 5), Health 2000 (n = 19), Helsinki Birth Cohort (n = 8), FINRISK 2007 (n = 8) and FINRISK 2012 (n = 13).

^c^P_trend_ value, two-sided test calculated using a continuous variable based on the median in each quintile.

^d^Multivariable model^a^ excluding ‘cohort’ as a confounding factor.

**Table 4:** Hazard ratios (HR) [Multivariable model was adjusted for age (years), education (tertiles by birth year), smoking (never, former, current smokers), height (cm, continuous), body mass index (kg/m^2^, continuous), leisure time exercise (passive, somewhat active, active), parity (never, ever), hormone replacement therapy (never, ever), energy intake (kJ/day, continuous) and cohort. The specific stratification factor was excluded from the model] and confidence intervals (95% CI) of postmenopausal breast cancer for dietary indices (Quintile 5 vs. Quintile 1) by the categories of body mass index and leisure time exercise.

	Number of cases	mNordic Dietary Index		mMediterranean Dietary Index		mAlternative healthy Eating (AHEI) index	
		Q5 vs. Q1 HR (95% CI)	P_between-categories heterogeneity_	Q5 vs. Q1 HR (95% CI)	P_between-categories heterogeneity_	Q5 vs. Q1 HR (95% CI)	P_between-categories heterogeneity_
**BMI, kg/m**^**2**^			0.48		0.22		0.74
< 25	87	0.70 (0.34–1.43)	–	0.72 (0.37–1.38)		0.87 (0.43–1.74)	
25–< 30	104	0.68 (0.33–1.40)	–	0.95 (0.50–1.82)		0.93 (0.48–1.81)	
≥ 30	83	0.75 (0.36–1.58)	–	1.04 (0.49–2.22)		0.89 (0.44–1.77)	
**Leisure time exercise**			0.85		0.14		0.29
Active	42	0.73 (0.28–1.93)	–	0.65 (0.23–1.78)	–	2.57 (0.81–8.17)	
Somewhat active	153	0.66 (0.37–1.18)	–	0.73 (0.44–1.23)	–	0.68 (0.39–1.16)	
Passive	79	0.82 (0.37–1.80)	–	1.50 (0.72–3.14)		0.95 (0.45–2.01)	
**Follow-up period, years**			0.67		0.48		0.49
< 5	126	0.78 (0.43–1.43)	–	1.06 (0.60–1.85)	–	1.05 (0.59–1.87)	
≥ 5	148	0.66 (0.37–1.17)	–	0.74 (0.43–1.29)		0.78 (0.46–1.34)

## Discussion

In this combined analysis of five population-based Finnish cohort studies, we observed a 33% decrease in postmenopausal BC risk for those with the highest compared to the lowest diet quality according to mNDI. The finding, however, was borderline significant (95% CI 0.48–1.01). Diet quality as measured by mMEDI and mAHEI was not associated with the risk of postmenopausal BC. These findings were not modified by categories of BMI or leisure time exercise, or by follow-up length. Furthermore, the specific components of the indices alone were not associated with the BC risk.

In a systematic review and meta-analysis of seven cohort studies from Sweden, United Kingdom, the Netherlands and ten European countries (EPIC) as well as the United States and Singapore (n = 675,954), high adherence to the Mediterranean diet decreased BC risk by 6% compared to low adherence (RR: 0.94, 95% CI 0.90–0.99)^4^. The Mediterranean diet especially decreased the risk of estrogen receptor-negative (ER−) postmenopausal BC, although not in all studies reviewed by Du et al.^34^. In more recent cohort studies, the scientific evidence for the Mediterranean diet, however, has been inconsistent. The California Teachers Study (n = 54,442 postmenopausal women) reported a borderline significant finding for the aMED index with (HR: 0.91, 95% CI 0.81–1.01, highest vs. lowest quintile) and without the alcohol component (HR: 0.91, 95% CI 0.82–1.02)^7^. In another US cohort study, no association was found in women who had a sister with BC but no prior BC themselves (The Sister Study, n = 50,884 women aged 35–74 years)^9^. Furthermore, the MEDI-LITE index was not associated with BC risk in the NutriNet Santé Cohort from France (n = 41,543 women aged 40 years and over)^8^. The inconsistent findings may be explained by the used MED indices that have not been completely consistent, different age distributions and populations or varying hormone receptor status of the tumours. The strongest evidence for an association between the Mediterranean diet and BC risk was observed in the Mediterranean countries^35,36^ and in EPIC study^37^. We did not find any association between mMEDI and postmenopausal BC risk in Finland. One explanation may be that women even in the highest quintile of the mMEDI score were possibly not following the true Mediterranean diet. Instead, our findings were most prominent with a borderline result for high adherence to the recommended Nordic diet assessed by the mNDI. In a cohort study from Sweden (n = 44,296 women aged 29–49 years), however, the Healthy Nordic Food Index (HNFI) including six food components (apples and pears; cabbages; root vegetables; whole-grain bread; oatmeal; and fish and shellfish) was not significantly linked with the risk of premenopausal or postmenopausal BC^10^.

Three previous US cohort studies have reported inconsistent results between AHEI (based on US dietary guidelines) and BC risk. In the California Teachers Study, AHEI was inversely associated with postmenopausal BC (HR 0.87, 95% CI 0.78–0.97, highest vs. lowest quintile)^7^. The result, however, was slightly attenuated (HR: 0.90, 95% CI 0.81–1.01) when the index was formulated without alcohol intake and alcohol was included as a confounding factor in the analyses. Instead, no associations were found for AHEI by the subtypes of BC tissue in the Nurses' Health Study (n = 100,643 women aged 35–55 years)^6^ or for the women with family history of BC^9^. Furthermore, a French cohort study did not observe a decrease in BC risk related to a healthy diet assessed by AHEI^8^. Our study supported those studies not finding high adherence to AHEI as a protective factor against postmenopausal BC. Furthermore, the interpretation of the findings remained the same when the alcohol component was excluded from each index but included as a confounding factor in the analyses.

In the meta-analyses of cohort studies, diets that scored high on the HEI (Healthy Eating Index), AHEI, and DASH (Dietary Approaches to Stop Hypertension diet) indices (combined) decreased the risk of all-cause mortality (reports n = 13), cardiovascular disease (n = 28), cancer (n = 31), type 2 diabetes (n = 10) and neurodegenerative disease (n = 5) by 22%, 22%, 16%, 18% and 15%, respectively^38^. In the analyses by cancer type, a decrease in risk of colorectal cancer (23%, n = 4), but not of BC (n = 2), was found. An umbrella review of meta-analyses (including 70 original studies) found inverse associations between the Mediterranean diet quality indices and the risk of type 2 diabetes, cardiovascular disease, cancer and cognitive-related diseases but the credibility of evidence was rated low to moderate^39^. Substantial heterogeneity among the MED indices was also observed. The healthy Nordic diet assessed by NDI, developed in our research group^26^, has been beneficially associated for instance with abdominal obesity^15^ weight gain^40^, cardiometabolic risk factors^41^, periodontal diseases^42^ and muscle strength^43^. Consequently, it seems that a healthy diet as a whole acts as a protective factor against many chronic diseases and their risk factors but it does not play a major role in the etiology of postmenopausal BC.

The strength of the study was its prospective data combining five Finnish cohort studies, which included extensive information about participants’ background, lifestyle and health. Furthermore, the cancer case data of the statutory Finnish Cancer Registry is reliable and comprehensive^30^. Another strength was that four of the cohort studies assessed the diet using the same validated FFQ^18–20^; the validated dietary history interview method was used in FMCF^17^.

The study was limited by the relatively small number of postmenopausal BC cases, which decreased the statistical power. We also used the age of 50 years as a cut-off point for postmenopausal status, because one cohort did not have information on the status. Consequently, some misclassification could exist between premenopausal and postmenopausal BC; however, only 16 cases were under 55 years old). Furthermore, each cohort study was planned independently, and thus, several characteristics (e.g., the age ranges and covariates) varied across the studies. However, we harmonized the classifications of covariates across the cohort studies and found no notable heterogeneity between them. Some classical risk factors for BC, however, could not be included as covariates in the statistical models, e.g., age at menarche and age at first birth, as they were missing in some of the cohorts. We do not have the information of emigration for these women. However, the effect of emigration from Finland is likely to be low. We also did not have information on hormonal receptor status of BC tissue (e.g., ER)^34^. The dietary methods often involve inaccuracies (e.g., under- or over-reporting), which may lead to biased results. These inaccuracies can be corrected to some extent by statistical means (e.g., energy adjustment), and the reliability/validity of used dietary methods have been found to be acceptable^17,18^. While diet was only assessed at baseline, the participant’s food consumption and nutrient intakes might have been misclassified if they changed markedly during the follow-up period. We assumed that measurement errors biased the results towards null. Additional analyses excluding BC cases diagnosed during the first two years of follow-up did not support presence of reverse causality due to undiagnosed cancer affecting eating habits. Finally, the results are not generalizable for young women as the etiology of premenopausal BC may differ from that of postmenopausal BC^3^. It is also good to keep in my mind that the findings may be underestimated due to health-conscious individuals being most likely to participate in health studies.

Although the findings from our combined data set seemed somewhat promising for the diet quality indices, especially for mNDI, these findings support the previous finding based on individual dietary factors that diet may not have a remarkable role in the development of postmenopausal BC. It seems that the most important risk factors for BC are related to ageing, hormonal and reproductive factors as well as body size, exercise and alcohol consumption. A challenge, however, in investigating BC is to define the most sensitive periods in a woman’s lifetime in terms of the development of the disease. For example, the interval between menarche and a first full-term pregnancy may be one of those life periods when breast tissue is more vulnerable to carcinogenic exposures^44^.

## Supplementary Information

Supplementary Information.

## Data Availability

The dataset used will be made available upon request through the Findata permit procedure. https://www.findata.fi/en/.
