# Supplementary material for: Association between overall diet quality and postmenopausal breast cancer risk in five Finnish cohort studies
Source: Sci Rep. 2021 Aug 18;11:16718. doi: 10.1038/s41598-021-95773-2 (PMC8373908; doi:10.1038/s41598-021-95773-2)
Supplement: Supplementary file 1 — Supplementary Information. [file 41598_2021_95773_MOESM1_ESM.pdf]

Article title: Association between overall diet quality and postmenopausal breast cancer risk in five Finnish cohort studies

Authors: Satu Männistö, Kennet Harald, Tommi Härkänen, Mirkka Maukonen, Johan G Eriksson, Sanna Heikkinen, Pekka Jousilahti, Niina E Kaartinen, Noora Kanerva, Paul Knekt, Seppo Koskinen, Maarit A Laaksonen, Nea Malila, Harri Rissanen, Janne Pitkäniemi
Corresponding Author: Satu Männistö, Finnish Institute for Health and Welfare,Finland, email: satu.mannisto@thl.fi

Supplementary Table S1. The specific food and nutrient components of diet quality indices used combined analyses.

**Index**	**Components**
**mNordic Dietary Index (mNDI)**	Fruits; vegetables; whole-grain products; low-fat milk; fish; red and processed meat; a ratio of polyunsaturated fatty acids to saturated fatty acids and trans-fatty acids; and alcohol.
**mMediterranean Dietary Index (mMEDI)**	Fruits; vegetables; legumes; nuts; whole-grain products; fish; red and processed meat; a ratio of monounsaturated fatty acids to saturated fatty acids; and alcohol.
**mAlternative Healthy Eating Index (mAHEI)**	Fruits; Vegetables; nuts and soy; whole-grain products; a ratio of white to red meat; a ratio of polyunsaturated fatty acids to saturated fatty acids; trans-fatty acids; and alcohol.

Supplementary Table S2. Consumption (median g/day) of specific components of diet quality indices of participants included in the combined analysis of the association between dietary indices and postmenopausal breast cancer risk.

	Cohort studies				
**Diet quality index components**	FMCF median (SD)	Health 2000 median (SD)	HBCS median (SD)	FINRISK 2007 median (SD)	FINRISK 2012 median (SD)
**Fruits**	150 (124)	212 (224)	245 (259)	282 (260)	211 (199)
**Vegetables**	76 (94)	247 (198)	273 (224)	322 (229)	267 (237)
**Legumes**	3 (5)	8 (15)	8 (11)	9 (16)	11 (21)
**Nuts and soya products**	0 (1)	0 (25)	0 (12)	1 (20)	3 (15)
**Whole-grain cereals**[a]	98 (63)	66 (38)	53 (37)	80 (49)	75 (46)
**Low-fat milk**	11 (234)	170 (239)	124 (231)	170 (241)	170 (246)
**Red meat and processed meat**	120 (78)	97 (86)	75 (66)	93 (81)	87 (68)
**Poultry**	0 (13)	13 (43)	24 (48)	20 (45)	21 (46)
**Fish**	17 (23)	40 (46)	39 (48)	40 (56)	47 (61)
**Saturated fatty acids**	40 (20)	31 (15)	25 (12)	25 (12)	27 (13)
**Monounsaturated fatty acids**	24 (10)	25 (12)	23 (10)	24 (11)	25 (12)
**Polyunsaturated fatty acids**	6 (4)	12 (6)	11 (5)	12 (6)	13 (6)
**Trans-fatty acids**	2 (1)	1 (1)	1 (0)	1 (0)	1 (0)
**Alcohol (100%)**	0 (5)	1 (4)	3 (7)	2 (6)	2 (6)

[a] Rye, oats and barley.
